# Editorial: Surveying Antimicrobial Resistance, Approaches, Issues, and Challenges to Overcome

**DOI:** 10.3389/fmicb.2017.00090

**Published:** 2017-02-01

**Authors:** Gilberto Igrejas, José L. Capelo, Alexandre Gonçalves, Patrícia Poeta

**Affiliations:** Functional Genomics and Proteomics Unit, Genetics and Biotechnology, University of Trás-os-Montes and Alto DouroVila Real, Portugal

**Keywords:** genomics, antimicrobial resistance, surveillance, molecular microbiology, epidemiology

The 1st International Caparica Conference in Antibiotic Resistance, was held in Caparica, Portugal in January 26–28, 2015. This very successful meeting attracted nearly 110 attendees, from 32 countries and involved a total of over 70 oral presentations, 16 shotguns presentations and 27 posters. The results and insights from this meeting are being made accessible to the general scientific community by this special dedicated issue of the Frontiers in Microbiology Research Topic.

Antimicrobial resistance within populations of different infectious agents is a worldwide public health threat. Already the available treatment options for common infections in some settings are becoming ineffective. There are now reports of bacterial resistance to all antibiotic classes used in either human or veterinary medicine, and in several cases, of an association between antibiotic use and the development of clinical resistance. To counter this emergent problem, the World Health Organization has appealed for urgent and concerted action by governments, health professionals, industry, civil society and patients to slow down the spread of drug resistance, limit its impact today and preserve medical advances for future generations.

The present Research Topic brings together a group of leading researchers from all over the world who have described different aspects of antimicrobial resistance patterns found in diverse ecosystems. The articles in this topic seek to address this question, including the epidemiology of resistance in animal and zoonotic pathogens, mobile elements containing resistance genes, the omics of antimicrobial resistance, emerging antimicrobial resistance mechanisms, control of resistant infections, establishing antimicrobial use and resistance surveillance systems, and alternatives strategies to overcome the problem of antimicrobial resistance worldwide.

We want to thank the reviewers for their many thoughtful and insightful comments, and the authors for their high-quality contributions. In closing, we would like to encourage all those who have not yet had a chance to participate in a “Caparica Meeting” and remind all to participate in the 2nd edition of the International Caparica Conference in Antibiotic Resistance that will be held in Caparica on 12–15th June 2017 (http://www.ic2ar2017.com).

## Author contributions

All authors listed, have made substantial, direct and intellectual contribution to the work, and approved it for publication.

### Conflict of interest statement

The authors declare that the research was conducted in the absence of any commercial or financial relationships that could be construed as a potential conflict of interest.

